# *QuickMIRSeq:* a pipeline for quick and accurate quantification of both known miRNAs and isomiRs by jointly processing multiple samples from microRNA sequencing

**DOI:** 10.1186/s12859-017-1601-4

**Published:** 2017-03-20

**Authors:** Shanrong Zhao, William Gordon, Sarah Du, Chi Zhang, Wen He, Li Xi, Sachin Mathur, Michael Agostino, Theresa Paradis, David von Schack, Michael Vincent, Baohong Zhang

**Affiliations:** 1 Early Clinical Development, Pfizer Worldwide Research and Development, Cambridge, MA 02139 USA; 2 Business Technology, Pfizer Worldwide Research and Development, Andover, MA 01810 USA; 3 I&I Research Unit, Pfizer Worldwide Research and Development, Cambridge, MA 02139 USA

## Abstract

**Background:**

Genome-wide miRNA expression data can be used to study miRNA dysregulation comprehensively. Although many open-source tools for microRNA (miRNA)-seq data analyses are available, challenges remain in accurate miRNA quantification from large-scale miRNA-seq dataset. We implemented a pipeline called QuickMIRSeq for accurate quantification of known miRNAs and miRNA isoforms (isomiRs) from multiple samples simultaneously.

**Results:**

QuickMIRSeq considers the unique nature of miRNAs and combines many important features into its implementation. First, it takes advantage of high redundancy of miRNA reads and introduces joint mapping of multiple samples to reduce computational time. Second, it incorporates the strand information in the alignment step for more accurate quantification. Third, reads potentially arising from background noise are filtered out to improve the reliability of miRNA detection. Fourth, sequences aligned to miRNAs with mismatches are remapped to a reference genome to further reduce false positives. Finally, QuickMIRSeq generates a rich set of QC metrics and publication-ready plots.

**Conclusions:**

The rich visualization features implemented allow end users to interactively explore the results and gain more insights into miRNA-seq data analyses. The high degree of automation and interactivity in QuickMIRSeq leads to a substantial reduction in the time and effort required for miRNA-seq data analysis.

**Electronic supplementary material:**

The online version of this article (doi:10.1186/s12859-017-1601-4) contains supplementary material, which is available to authorized users.

## Background

MicroRNAs (miRNAs) are a class of endogenous small (about 22 nucleotides (nt)) non-coding RNAs that play important roles in the regulation of gene expression. The miRNA genes are first transcribed as primary miRNAs that are further processed into pre-miRNAs by Drosha, an RNase III enzyme [1–3]. Then pre-miRNAs are exported to the cytoplasm and processed by Dicer, another RNase III enzyme, to generate a ~22-nt duplex consisting of a mature miRNA and its corresponding star miRNA [4, 5]. Finally, the duplex is unwound to give rise to mature miRNAs. Mature miRNA species may be generated from the 5′ and/or 3′ arms of the precursor duplex, and are called miRNA-5p and -3p, respectively. The mature miRNA is incorporated into a miRNA-induced silencing complex (miRISC), which then binds to the 3′-UTR of the target mRNA transcript, leading to translational inhibition or mRNA degradation.

The significance of miRNAs in health and disease is still an unfolding story. A single miRNA can regulate hundreds of target mRNAs concurrently. Importantly, aberrant regulation of miRNAs plays a central role in pathological events underlying cancers [5] and neurodegenerative diseases [6, 7]. Many researchers have demonstrated the potential role of miRNAs as non-invasive biomarkers of a variety of diseases [8–11]. Targeting miRNAs provides an emerging opportunity to develop effective miRNA-based therapy [12]. The rising body of advanced preclinical evidence on the biological significance of miR-221/222 in a variety of malignancies indicates that they will play a crucial role in the future of innovative therapeutic strategies, both as validated biomarkers and drug targets [13].

Recent advances in next-generation sequencing (NGS) technologies have enabled the interrogation of genome-wide miRNA expression at high throughput and low cost [14–17]. Deep sequencing of miRNA (miRNA-seq) has provided researchers an opportunity to catalogue the repertoire of miRNA expression across various tissues and models and comprehensively study their dysregulation. Importantly, miRNA profiling by sequencing can better distinguish very similar miRNAs compared with other available methods, including microarrays and qPCR panels. The NGS approach is a powerful way of cataloguing miRNAs, and has led to an exponential increase in miRBase entries in the last few years [18].

Many groups have developed open-source tools for miRNA-seq data analysis, including mirTools [19], DSAP [20], miRNAkey [21], miRanalyzer [22], miRDeep2 [23], miRExpress [24], UEA sRNA workbench [25], sRNAtoolbox [26], miRspring [27], iMir [28], Oasis [29], iSRAP [30], CAP-miRSeq [31], and miRge [32]. These tools differ in the methods and algorithms used for various processing steps such as adapter trimming and sequence alignment. Despite the availability of these tools, many bioinformatics challenges remain. On the one hand, a miRNA-seq dataset is enriched for small RNA species between 19 and 23 nt, and short sequence lengths make it more likely that a read maps to a genomic locus or known miRNA simply by chance in a large and complex reference genome. On the other hand, a sequence read can map to more than one miRNA, and how to deal with multiple mapping reads is still a challenge. This issue becomes more severe when miRNA-seq reads derived from multiple precursors are aligned to a reference genome directly. Therefore, for accurate miRNA quantification, it is especially important to introduce computational strategies to reduce or minimize potentially false mappings.

Nearly all miRNA-seq data analyses are performed using Linux clusters or workstations. However, analysis results in Linux are often hard to access for most bench scientists. Moreover, analyses of miRNA-seq datasets typically generate large amounts of data and a variety of result files that are difficult to interpret. Therefore, it is crucially important to organize and share miRNA-seq data analysis results in an efficient and user friendly way. Interactive web interfaces that allow end users not only to navigate all the quality control (QC) metrics and quantification results, but also to drill down and gain more insights into miRNA-seq datasets are thus much preferred.

To address those aforementioned challenges, we implemented a pipeline called QuickMIRSeq to advance accuracy, efficiency, and automation of miRNA-seq data analysis to the next level. QuickMIRSeq is, in part, motivated by our development of QuickRNAseq [33], an integrated tool for large-scale RNA-seq data analyses. QuickMIRSeq reconciles its implementation with the unique nature of miRNAs. Specifically, we require that QuickMIRSeq would:

1. group miRNAs with identical or similar sequences to solve or mitigate the multiple mapping issue of sequencing reads;
2. be strand-aware, and respect the fact that miRNA-seq dataset are intrinsically sense stranded;
3. implement joint mapping of multiple samples for both computational efficiency and filtering out noisy background reads to improve the reliability of miRNA detection and quantification;
4. remap those sequences with mismatches to known miRNAs to the reference genome to further reduce potentially false positives;
5. quantify the expression levels of both miRNAs and isomiRs; and
6. organize results in a user-friendly manner, make them fully accessible via a web interface, and enable end users to interactively digest analysis results in a user friendly manner.

## Implementation

**Fig. 1:**
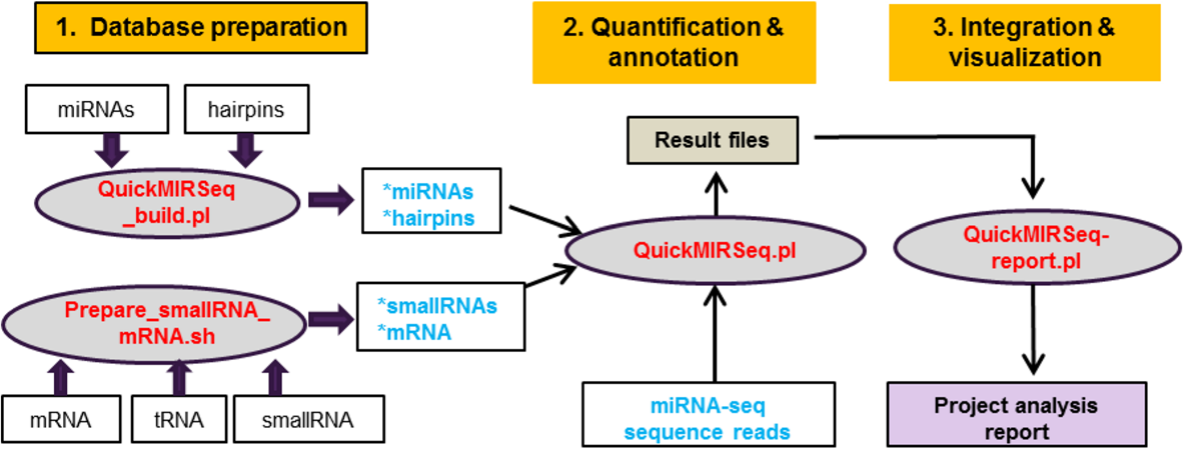
Overview of the QuickMIRSeq pipeline

QuickMIRSeq is designed for quick and accurate quantification of known miRNAs and isomiRs from large-scale small RNA sequencing, and the entire pipeline consists of three main steps (Fig. 1), i.e. (1) database preparation, (2) quantification and annotation, and (3) integration and visualization. Step #1 prepares databases required for Step #2; Step #2 processes the miRNA-seq dataset and generates count tables for miRNAs and isomiRs; and Step #3 produces an integrated and interactive project report for data analyses. Step #1 requires to run only once for any given species, and then the databases can be shared by many miRNA-seq projects. Steps #2 and #3 are accomplished by Perl scripts *QuickMIRSeq.pl* and *QuickMIRSeq-report.pl*, respectively.

### Step #1: Database preparation

**Fig. 2:**
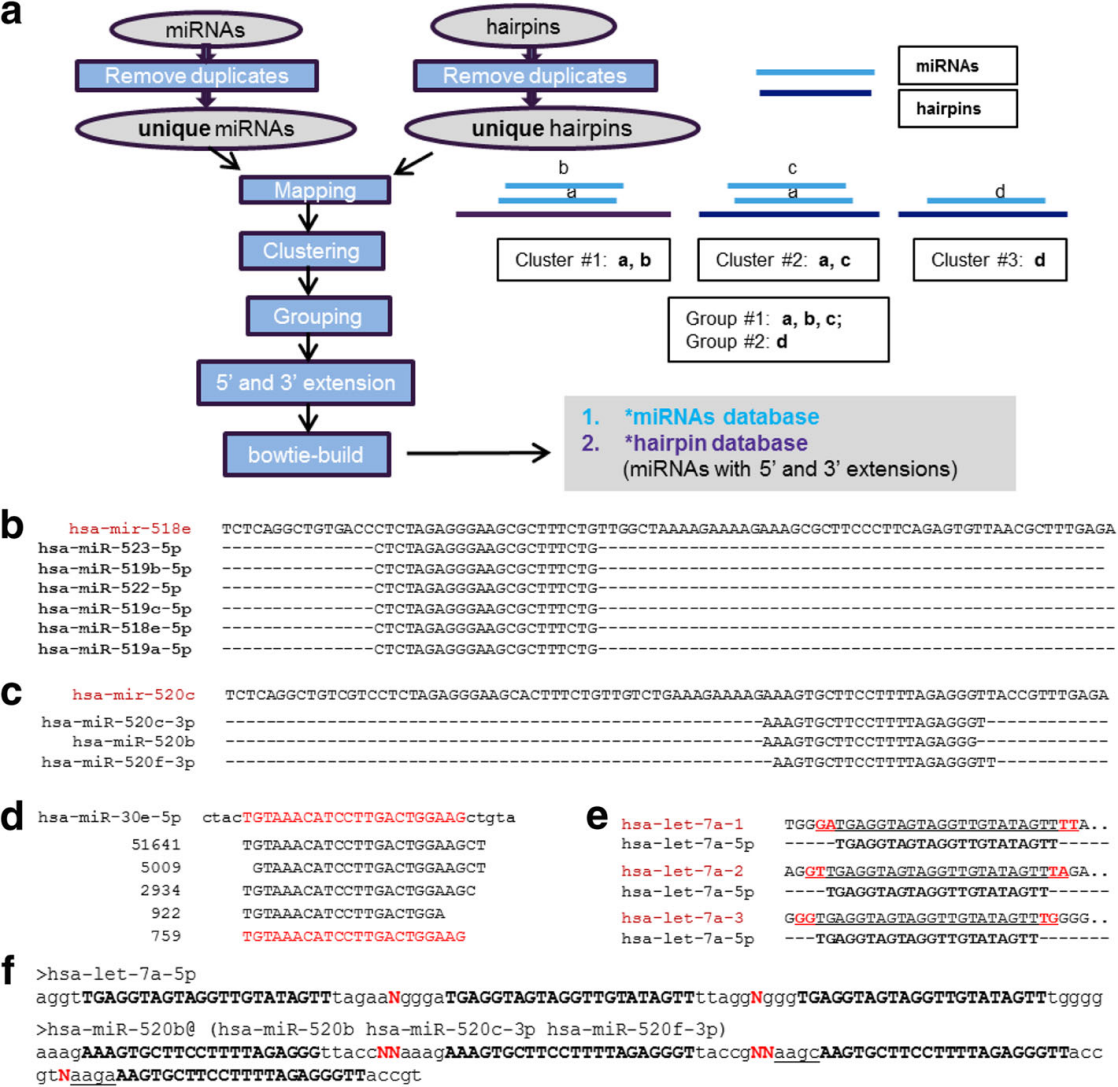
Database preparation. **a** The flowchart of creation of modified miRNA and hairpin database. **b** Mature miRNAs with identical sequences. **c** Sequences from a model miRNA cluster; i.e., different miRNAs mapping (and overlapping) to a region of the same hairpin precursor. **d** The same pre-miRNA gene loci can generate many miRNA isoforms, but the most abundant isoform in a particular sample may not necessarily be the one annotated in miRBase. The numbers in the first column represent counts of sequences identical to the sequences in the second column. **e** A mature miRNA can be derived from more than one precursor. **f** Representative entries in a *miRNA database. All extended bases are in lower case, while the nucleotides that correspond to mature miRNAs are in upper case

This step prepares miRNA, hairpin, and small RNA and mRNA databases for Step #2. Two scripts were written to automate the entire step (Fig. 1). The utility script *Prepare_smallRNA_mRNA.sh* is used to prepare small RNA and mRNA databases. It automates the downloading mRNA, tRNA, and small RNA database from a variety of public domains, performs cleanup and extraction, and generates bowtie index libraries for small RNA and mRNA. *QuickMIRSeq_build.pl* takes a mature miRNA and a hairpin sequence database in FASTA format as input, and creates modified miRNA and hairpin databases for alignment in Step #2. The workflow for creating the modified miRNA and hairpin databases is depicted in Fig. 2a, and the main issues to be addressed are described as follows.

All known mature miRNAs and hairpins can be downloaded directly from miRBase [18]. Currently, there are 1881 precursors and 2588 mature miRNAs (human) registered in the most recent miRBase release 21. Some mature miRNA sequences are identical (see the example in Fig. 2b), though derived from different pre-miRNA sequences (Additional file 1: Figure S1). Examples of such miRNA sequences are found in the same cluster on human chromosome 19. Presumably, a single gene was expanded to form all the other paralogs. The paralogs have maintained the same mature miRNA sequence, but their genomic sequences have slowly diverged, potentially leading to alternate functions. Therefore, the redundant sequence entries in miRNA and hairpin databases are first removed, and only unique sequences are kept. The identical miRNA and hairpin groups are listed in Additional file 1: Table S1 and Additional file 1: Table S2, respectively. Next, unique miRNAs are mapped to unique hairpins. Individual miRNA clusters are identified, and then merged into groups if any two clusters share a common miRNA member. Although miRBase registers hsa-miR-520c-3p, hsa-miR-520b, and hsa-miR-520f-3p as three separate mature miRNAs, they are all aligned to the same hairpin precursor in Fig. 2c. If the alternative splicing events of 5′ and 3′ end are taken into account, the sequence reads derived from these three miRNAs are nearly indistinguishable. In QuickMIRSeq, we group overlapping mature miRNAs mapped to the same precursor into individual clusters.

Another issue in quantification of miRNAs is the presence of isomiRs (see Fig. 2d). Unfortunately, miRBase annotates only one mature miRNA for a given miRNA locus, and often the most abundant isomiR present in the sample is not necessarily the one annotated in miRBase [34]. For instance, the most abundant hsa-miR-30e-5p isoform in Fig. 2d is 2 bp longer at the 3′ end than the miRBase annotation. Therefore, it is not sufficient to use only annotated miRNAs in miRBase as the sole reference for accurate miRNA quantification. To capture the entire set of isomiR length variants, all the annotated miRNA sequences are extended at the 5′ and 3′ ends by adding user specified additional nucleotide bases from their corresponding hairpin precursors. The extended miRNAs are used in the sequence alignments.

More than 50 mature miRNAs are found in two or more hairpin precursors in the human genome [35], and hsa-let-7a-5p is used to exemplify this point (see Fig. 2e). These loci produce identical mature miRNAs but often have different nucleotides adjacent to the mature sequence. Accordingly, after the 5′ and 3′ end extension, one mature miRNAs can generate more than one extended sequence. These extended sequences are combined to represent hsa-let-7a-5p (see Fig. 2f) in the database. It is noted in Fig. 2f that the extended nucleotides are in lower case while mature miRNA sequences are in upper case. Different extended sequences are delimited by either a single “N” if corresponding to the same mature miRNAs, or a double “NN” if corresponding to different mature miRNAs.

### Step #2: Quantification and annotation

**Fig. 3:**
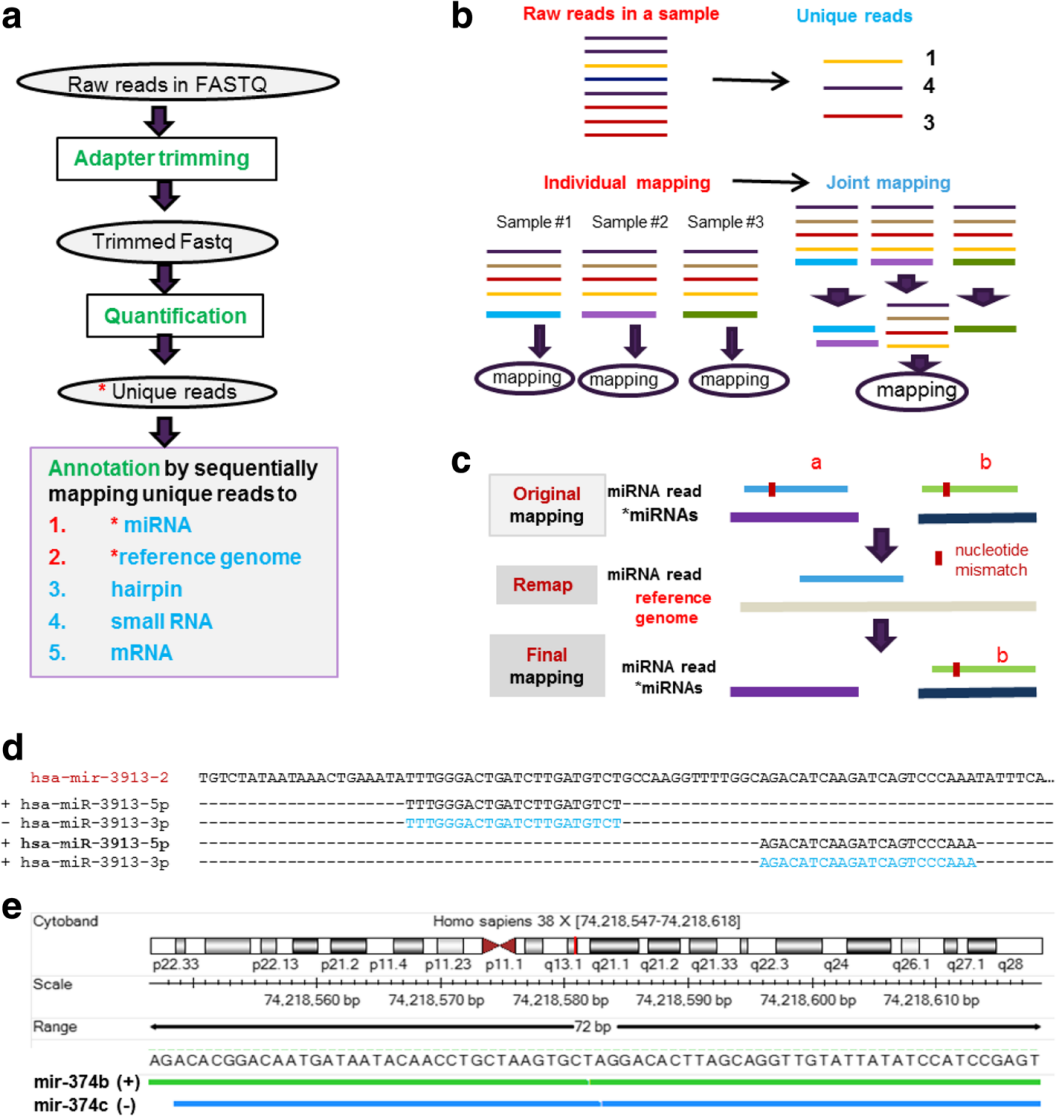
Various strategies introduced in QuickMIRSeq to reduce computational time and to improve accuracy in miRNA quantification. **a** The overview of workflow for miRNA-seq data analysis. **b** Collapsing identical reads into unique ones within and across multiple samples. **c** Remapping of miRNA reads with mismatches to the reference genome to reduce false hits. **d** miRNAs has-miR-3913-5p and has-miR-3913-3p are generated from the same hairpin precursor and reverse complementary to each other. **e** Genes hsa-mir-374b and hsa-mir-374c are expressed from the same genomic locus in chromosome X but transcribed in opposite directions

Figure 3a outlines the main flowchart for Step #2. First, all adapter sequences are trimmed from raw sequencing reads, and then short miRNA-seq reads are collapsed into unique reads as illustrated in Fig. 3b within and across samples. Next, the unique reads are mapped sequentially to the miRNA, hairpin, small RNA, and mRNA sequence databases prepared in Step #1. Our implementation exploits as many unique features of miRNA reads as possible, and a variety of strategies are introduced for computational efficiency and accuracy in quantification. These include collapsing identical reads into unique ones and joint mapping of unique reads across multiple samples (Fig. 3b), remapping of miRNA reads with mismatches to the reference genome to reduce false hits (Fig. 3c) and taking into account the strand information for more accurate read mapping (Fig. 3d and e).

A major feature of miRNA reads that can be utilized is their high redundancy. Therefore, collapsing identical reads into unique ones is advantageous in miRNA-seq data analysis because it significantly reduces the number of miRNA reads in the alignment step. A step that collapses identical reads has been adopted by miRDeep2 [23], miRExpress [24], sRNAbench [26], miRge [32], and other programs. A miRNA-seq study often consists of many samples from different biological conditions. It is expected that the majority of identical miRNA reads can be found in most samples of a given dataset. Instead of processing individual samples independently, it is more efficient to perform an alignment using the combined unique sequence reads identified across multiple samples (Fig. 3b).

Short reads derived from genomic loci can be mapped to miRNAs by chance, especially when mismatches are allowed. Therefore, QuickMIRSeq introduces an optional “Remapping” step to map those sequences with mismatches to the reference genome to reduce the number of likely false positives (Fig. 3c). If a mismatch read can be mapped perfectly to the reference genome without any mismatch, its mapping to the miRNA will be invalidated. Read “a” in Fig. 3c is a case in point. The mapping of Read “b” is kept because no perfect mapping is found in the reference genome.

In all current small RNA sequencing protocols, the adapters are ligated first to the RNA molecule, and therefore miRNA-seq dataset are intrinsically stranded. However, bowtie [36] by default will attempt to align a sequence read against both the forward and reverse-complement reference strands, and this is problematic as illustrated in Fig. 3d and e. As shown in Fig. 3d, mature miRNA species can be generated from both the 5′ and/or 3′ arms of the same hairpin precursor, and these two miRNAs can even be reverse complementary to each other, such as hsa-miR-3913-5p and hsa-miR-3913-3p. In this scenario, reads derived from hsa-miR-3913-5p can be mapped to the reverse-complement strand of hsa-miR-3913-3p, and vice versa. In Fig. 3e, hsa-mir-374b and hsa-mir-374c are expressed from the same locus in chromosome X but transcribed in opposite directions. Likewise, reads derived from this locus become ambiguous if the strand information is ignored in the alignment step. More miRNA pairs that are reverse complementary to each other are listed in Additional file 1: Table S3. If the strand information is ignored, the accurate quantification becomes problematic for those miRNA pairs in Additional file 1: Table S3. Thus, it is crucial to specify *“--norc*” option to instruct bowtie not attempt to align against the reverse-complement reference strand when analyzing currently sequenced miRNA-seq dataset.

The joint mapping procedure was first introduced by miRge [32]. QuickMIRSeq not only incorporates this strategy into its alignment step, but also extends it to filter out potentially noisy background reads to improve the reliability of detected miRNAs. In our experience, sufficient sequencing depth reveals low expressing miRNAs (true positives) across many samples, while noisy background reads (false positives) are more likely to be seen only in a very small subset of samples. Therefore, potentially noisy reads can be identified based on the patterns of their read counts across samples and accordingly filtered out. As we will show later, the filtering of noisy reads barely impacts the total number of mapped miRNA reads, but significantly reduces the number of detected miRNAs.

Previously, each arm of the hairpin precursor miRNA is believed to give rise to a single mature product. However, recent advances have revealed that a number of distinct mature miRNA species can arise from the same hairpin arm, and thus significantly increase the diversity and complexity of the miRNAs. Recent additional studies have shown that isomiR sequences are tissue and gender-specific [34] and play distinct roles in biological processes [37], which emphasize the importance of performing miRNA-seq analysis simultaneously at both the miRNA and isomiR levels. To this end, the QuickMIRSeq pipeline produces parallel quantification results for miRNAs and isomiRs. The protocol for isomiR quantification is detailed in Additional file 1: Figure S2. In brief, the 5′ and 3′ end offsets for all mapped reads are identified first. Then reads that have identical 5′ and 3′ end offsets are added up to generate an isomiR counts table.

Quite often, end users are required to make an uninformed choice in advance between inclusion and exclusion of sequences that contain mismatches when analyzing miRNA-seq datasets. If end users change their minds, the same dataset have to be re-analyzed. To help end users to make an informed decision afterwards, the QuickMIRSeq pipeline generates companion counts tables in which only sequences with mismatches are counted, in addition to the ‘standard’ counts table for miRNA and isomiRs. The companion tables serve two purposes. First, they can be used for quality controls and secondly, they offer the end user a choice of using only perfectly matched reads for downstream analysis, eliminating the need to reanalyze the entire dataset. Instead, the subtraction of the companion counts table from its ‘standard’ miRNA or isomiR counterpart provides the necessary information.

### Step #3: Integration and visualization

**Fig. 4:**
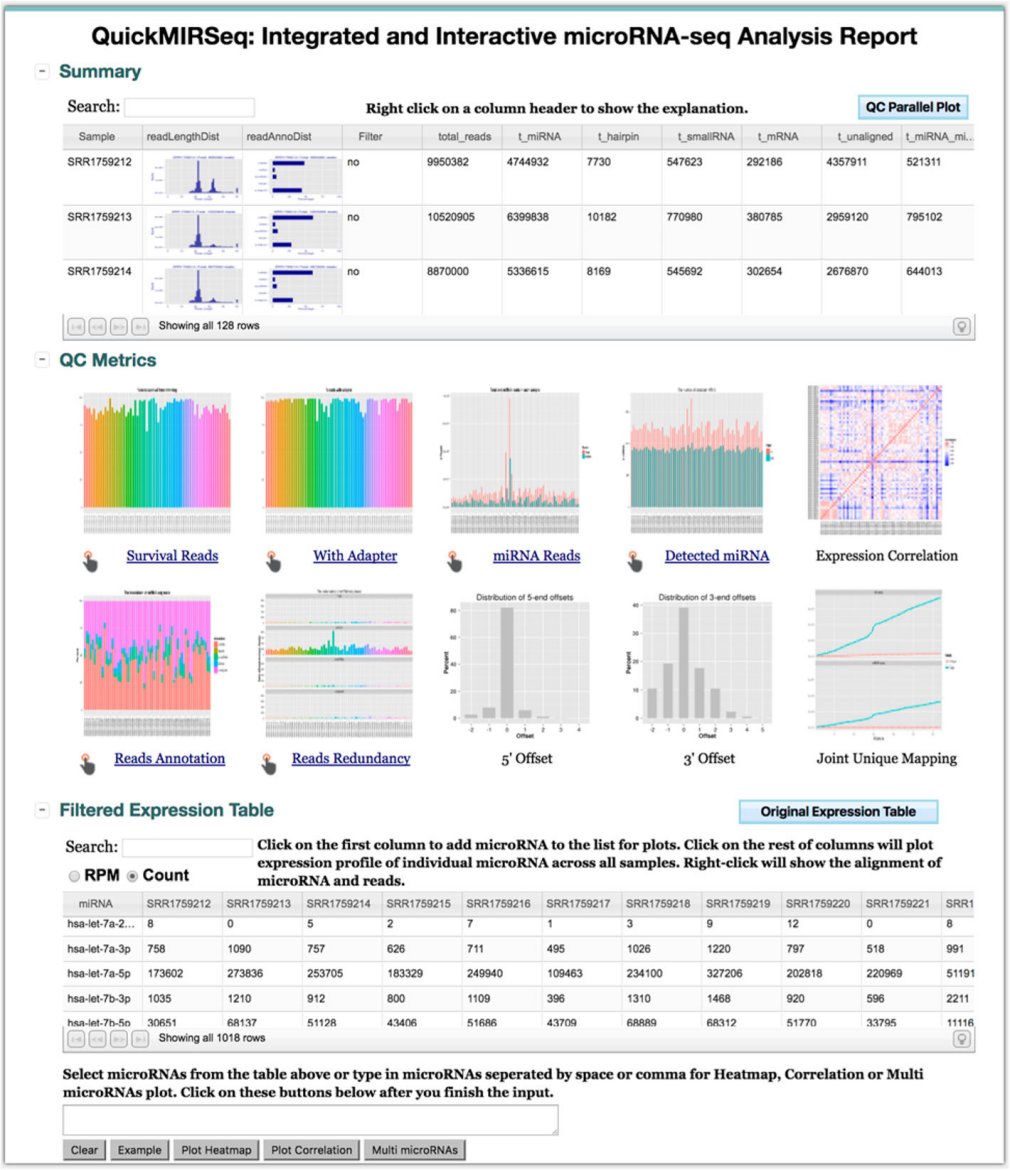
Entry page of a project report. The first section provides the summary of reads mapping and annotation for individual samples. The second section shows a variety of key QC metrics, including adapter trimming, detected miRNAs and distributions of variation at 5′ and 3′ ends of miRNA reads. The third section lists expression values of detected miRNAs in each sample, which can be interchangeably shown as either raw counts or RPMs on the fly

This step automates the generation of various QC plots and produces an integrated interactive project report. All high-quality plots are ready for PowerPoint presentation and scientific publications. From the entry webpage of the project report as shown in Fig. 4, a user can easily navigate and visualize analysis results. More importantly, the project report offers interactive visualizations of miRNA-seq QC and expression results. The visualization in QuickMIRSeq is implemented by combining cutting edge JavaScript-based open source visualization libraries, including JQuery, D3 (Data-Driven Documents), canvasXpress, and Nozzle [38]. JQuery simplifies HTML page traversal, manipulation, event handling, and animation, while D3 can manipulate HTML documents based on input data. Nozzle [38] is designed to facilitate summarizing and rapid browsing of complex results in data analysis pipelines when multiple analyses are performed on big datasets. All required JavaScript libraries have already been packaged into the QuickMIRSeq project report; thus, the report can be digested on a PC locally and deployment into a web server is optional.

## Results

QuickMIRSeq can analyze miRNA-seq datasets from any species as long as the corresponding mature miRNA and hairpin databases are available. We selected three datasets GSE64977 [7], GSE65920 [39], and GSE60900 [40] for test runs, corresponding to human, mouse and rat, respectively. All three datasets were generated and deposited into GEO between 2014 and 2016. The complete project reports can be downloaded from the QuickMIRSeq project home page (http://QuickMIRSeq.sourceforge.net). We will use the GSE64977 dataset to highlight important functionalities and features of QuickMIRSeq. All the results presented below, including summaries and QC plots, were generated automatically by the QuickMIRSeq pipeline, and end users are not required to perform any additional analysis steps.

### Integrated and interactive project report

A screenshot of the entry webpage for the project report is shown in Fig. 4. The page consists of three main sections. The first section provides the summary of reads mapping and annotation for individual samples, including the distribution of read lengths, the breakdown of read annotations, and the number of reads falling into miRNA, hairpin, small RNA, mRNA and unaligned categories, respectively. Clicking on “QC Parallel Plot” button will show an integrated and interactive QC plot for linked quality control measurements. The second section shows an array of key QC metrics graphically, including adapter trimming, detected miRNAs and distributions of variation at 5′ and 3′ ends of miRNA reads. For each QC plot, clicking on the icon image will bring forth the corresponding enlarged plot, and the interactive plot is accessible by clicking the pointing hand. The third section lists expression values of detected miRNAs in each sample, which can be interchangeably shown as either raw counts or RPMs (Read Per Million).

### Incorporation of strand information gives more accurate quantification

**Fig. 5:**
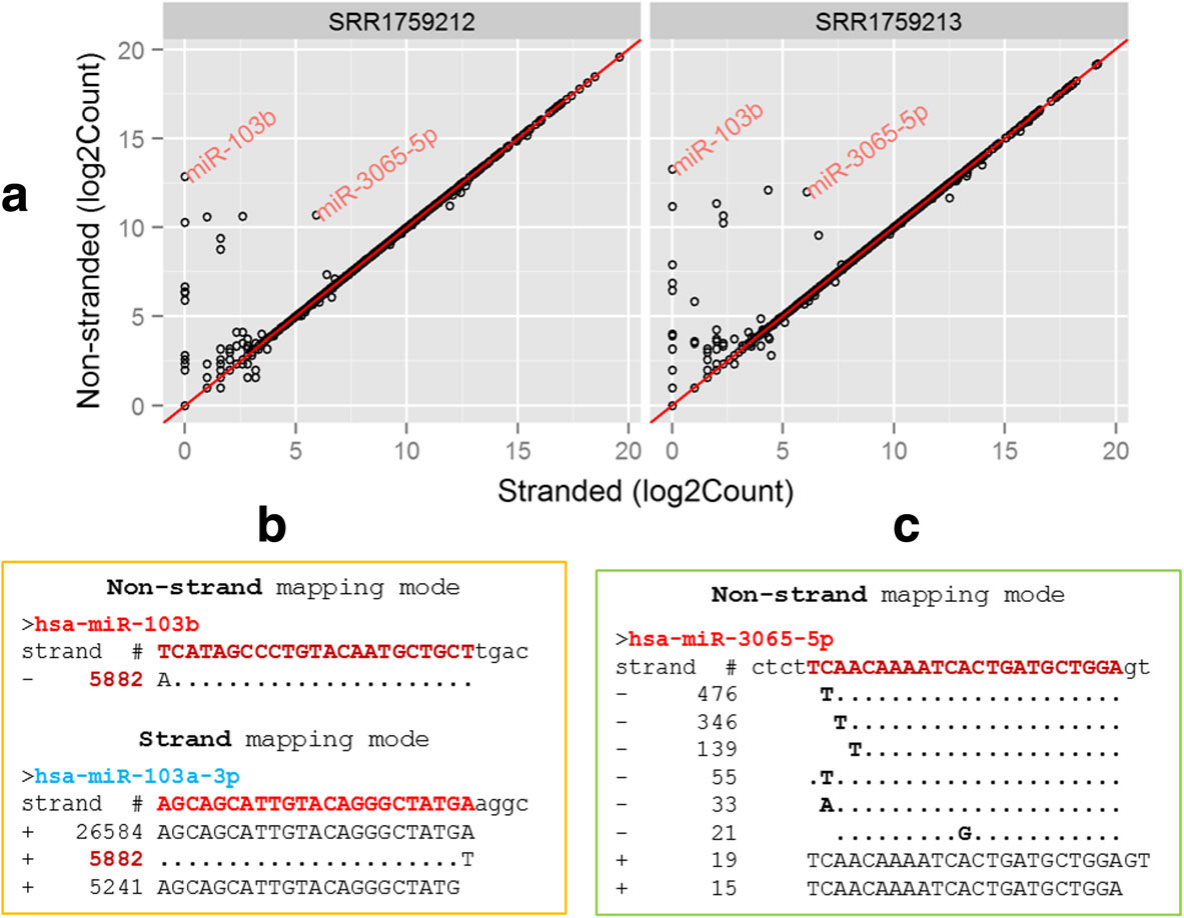
Incorporation of strand information gives more accurate quantification. **a** Scatter plots of miRNA quantification results. **b**,**c** Many reads are wrongly mapped to anti-sense strand of has-miR-103b and has-miR-3065-5p when the stranded information is ignored in miRNA-seq quantification. Samples SRR1759212 and SRR1759213 are stranded, but analyzed with and without incorporation of the strand information, respectively

It was demonstrated more accurate quantification is obtained in stranded mRNA-seq than in non-stranded mRNA-seq [41]. To demonstrate the importance of the strand information in miRNA quantification, four samples (SRR1759212, SRR1759213, SRR1759214, and SRR1759215) in the dataset were analyzed by the QuickMIRSeq pipeline with and without incorporation of the strand information in the alignment, and the scatter plots for SRR1759212 and SRR1759213 are shown in Fig. 5a. In comparison, the four samples were also analyzed using miRge [32], and the results are shown in Additional file 1: Figure S3. The comparison between QuickMIRSeq and miRge is discussed later in a separate section. The majority of miRNAs are arrayed along the diagonal line in the scatter plots and their quantification results are either identical or very close. However, there are some miRNAs whose quantifications are influenced dramatically by the strand information. Additional file 1: Table S4 lists the top 10 miRNAs with large differences. To better understand the reasons for the observed large differences, miR-103b and miR-3065-5p were selected for in-depth analysis.

As shown in Fig. 5b, the sequence *AGCAGCATTGTACAGGGCTATGT* has 5882 copies in SRR1759212. If the strand information is ignored, this read maps equally well to the sense strand of hsa-miR-103a-3p and to the antisense strand of hsa-miR-103b. In fact, the dataset is sense-stranded, and thus the mapping to hsa-miR-103a-3p is true, whereas the alignment to hsa-miR-103b is wrong. Fig. 5b shows that ignoring the strand information underestimates the expression of one of the miRNAs and overestimates the other. In Fig. 5c, a large number of reads were aligned to the antisense strand of hsa-miR-3065-5p; however, these reads would not be mapped if the stranded sequencing protocol was taken into consideration. As a result, the expression of hsa-miR-3065-5p is overestimated due to false mappings. Therefore, incorporation of strand information in the alignment step gives rise to more accurate quantification.

### Benefits of joint mapping and remapping of mismatch reads

**Fig. 6:**
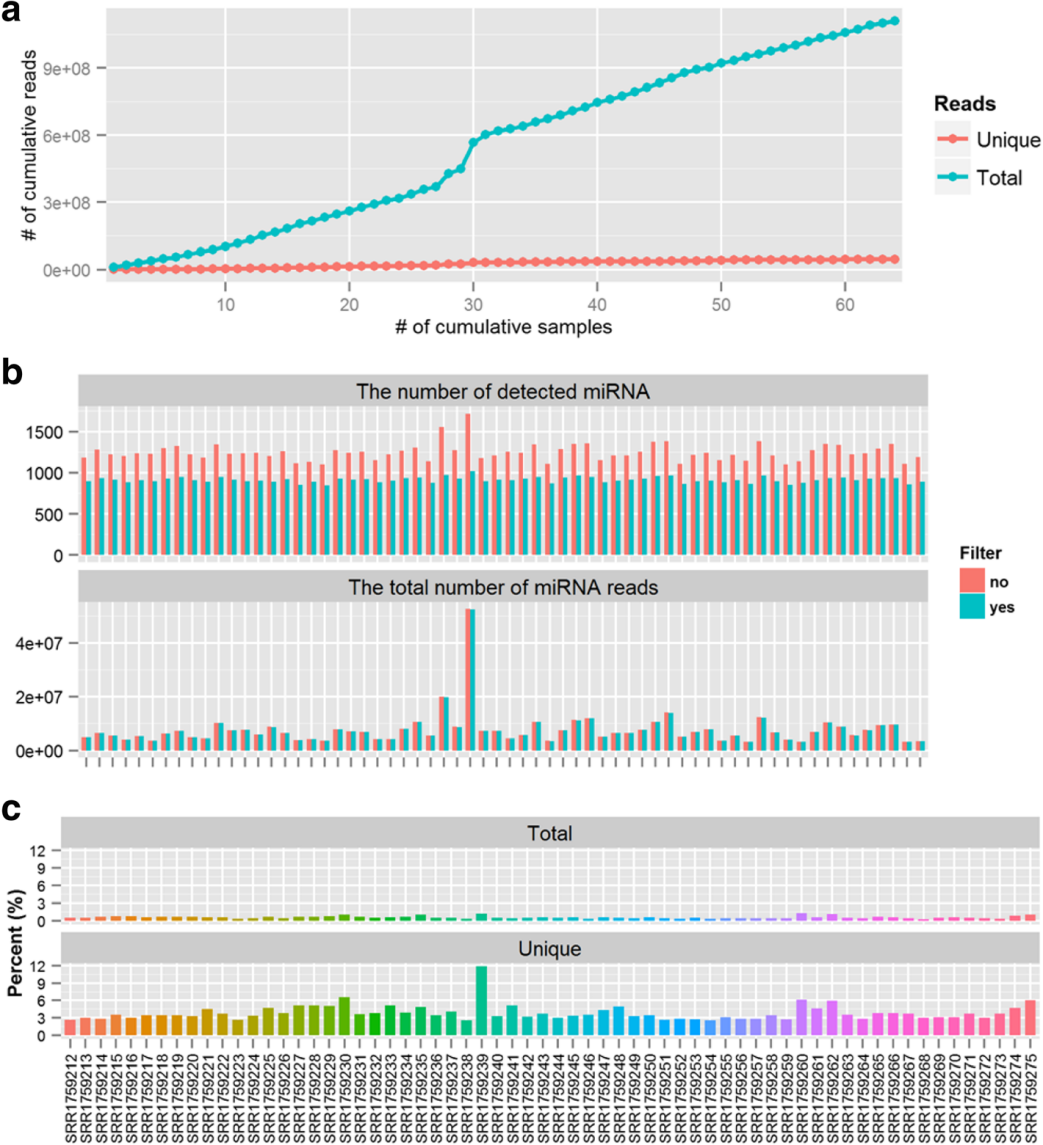
The benefits of joint mapping and “Remapping” of mismatch reads. **a** If individual samples are processed independently, a total of 1,110,470,294 sequences need to be aligned. This number drops to 47,355,430 if all 64 samples are jointly processed. **b** The filtering of noisy reads significantly reduces the number of detected miRNAs (Top panel) but barely impacts the total number of mapped miRNA reads (Bottom panel). A read is filtered out if it has ZERO counts in more than 60% of samples, or its average count across all samples is below 2. **c** For unique mismatch reads, the average invalidation rate is 3.8% (ranging from 2.7 to 6.1%); however, the corresponding average rate is only 0.64% for all mismatched reads. Note the y-axis indicates the percentage of miRNA reads that are invalidated in the “Remapping” step

For GSE64977, if each individual sample is processed independently, a total of 1,110,470,294 reads need to be aligned, whereas this number drops to 47,355,430 if all 64 samples are combined and jointly processed (Fig. 6a). The benefit of joint mapping becomes increasingly evident as the number of sample increases. The strategy of joint mapping of multiple samples takes advantage of the high redundancy of miRNA-seq reads within and across samples, and it significantly reduces computational time. In our HPC cluster, it took bowtie (with 8 running threads) 0.57 h to align 47 million reads to miRbase, and the time would rise to 13.3 h if mapping all 1.11 billion raw reads without introducing the strategy of jointing mapping. In the meantime, joint mapping is powerful in filtering out false positives (i.e., noisy reads), thereby improving the reliability of the detected miRNAs (Fig. 6b). As depicted in Fig. 6b, the filtering of noisy reads barely impacts the total number of mapped miRNA reads, but significantly reduces the number of detected miRNA, and accordingly, improves the statistical power in downstream differential analysis of miRNAs. In Fig. 6b, a read is considered noisy if it is absent in more than 60% of the samples, and the average number across samples is less than two. In the QuickMIRSeq pipeline, end users can define the criteria for noisy reads when analyzing their miRNA-seq datasets.

The motivation of remapping reads with mismatches to the reference genome is to reduce potential false hits. The impact of “Remapping” on miRNA reads with mismatches is shown in Fig. 6c. The invalidation rate was calculated for all mismatch reads and unique mismatch reads, respectively. For unique mismatch reads, the average invalidation rate was 3.9% (ranging from 2.8 to 11.8%). However, the corresponding average rate was 0.61% for all mismatch reads. This confirms that the majority of the invalidated reads has low abundance and thus, is most likely to be false positives. Clearly, the invalidation rate varies greatly from sample to sample (Fig. 6c). This “Remapping” is implemented as an optional step, although it is recommended for miRNA-seq data analyses.

As mentioned in the Implementation section, the QuickMIRSeq pipeline separates the mapped reads into two categories: perfect and mismatches, and generates a companion counts table for reads with mismatches only, in addition to the standard counts table. On average, about 12% (ranging from 9 to 17%) mapped reads have mismatches (see Additional file 1: Figure S4). In general, we recommend that reads with mismatches are included in the quantification step because their exclusion can underestimate miRNA expression levels.

### Comprehensive analysis reports and rich QC metrics

**Fig. 7:**
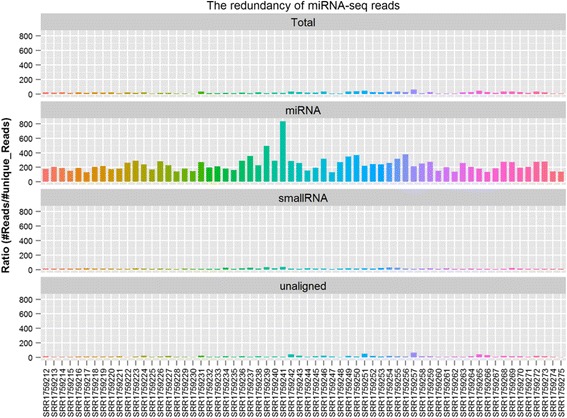
Read duplications in each annotated RNA category. As expected, the redundancy in miRNA reads is typically high, while redundancies in other annotated categories are generally much lower. The sample names from the GSE64977 miRNA-seq dataset used in this study are shown along the X-axis

As shown in Fig. 3a, adapter-trimmed reads undergo four separate alignments against miRNA, hairpin, small RNA, and mRNA sequences in a sequential manner. After annotation, QuickMIRSeq provides an overview of the distribution of annotated reads in each sample (Additional file 1: Figure S5). The relative abundance of annotated reads in each category is sample dependent. Usually, only a tiny portion of reads are mapped to hairpins. For high-quality miRNA-seq datasets, miRNAs should be dominant compared with other annotated categories. Furthermore, dividing the total number of reads by the unique number of reads gives rise to the read redundancy in each annotated category (Fig. 7). Depending on sequencing depth, the redundancy for miRNA reads can be as high as several hundred folds, whereas the redundancies of reads in other annotated categories are generally much lower. This feature can be used to identify potential issues in sequencing samples. For example, when we analyzed an in-house cell-free miRNA-seq dataset from urine, we found some samples had exceptionally high redundancy in unaligned reads (unpublished data). It turned out that many unaligned reads in those samples resulted from dimerization of primers added during the library preparation step. Because the amount of RNA in cell-free urine is low, this is more likely to happen than in other sample types. Therefore, the redundancy plot (shown in Fig. 7) is very helpful in trouble-shooting potential issues arising from difficult samples during library preparation.

The sequence read length in most miRNA-seq datasets that we analyzed was 50 bp, much longer than the typical length of a miRNA (20–23 bp). All adapter sequences need to be trimmed prior to alignment. After trimming, reads that are shorter than 16 bp are discarded and excluded from further analysis. QuickMIRSeq automatically generates a summary QC report for adapter trimming step (see Additional file 1: Figure S6). Ideally, the percentage of reads with adapter sequences should be close to 100% in a high-quality miRNA-seq dataset (Top panel). The trimmed reads should still be long enough to be kept for alignment, and thus the percentage of reads surviving adapter trimming should also be very high (Bottom panel). Moreover, a high-quality miRNA-seq dataset is expected to have a characteristic read length distribution. For human samples, the length distribution of the trimmed reads should, in principle, be centered on 22 bp. The read length distributions for samples SRR1759212, SRR1759213, SRR1759214, and SRR1759215 from GSE64977 are shown in Additional file 1: Figure S7. The peak is indeed at 22 bp for all four samples, and the overall pattern of length distribution indicates the majority of reads are derived from miRNAs. Usually, the length distribution is very informative on miRNA-seq data quality.

The offsets of all unique reads are first calculated with respect to miRNA seed sequences. Next, the distribution of offsets is examined and depicted as shown in Additional file 1: Figure S8 (see also Additional file 1: Table S5). Generally speaking, the 5′ end shows a much narrower range (+/−1 nt) of variations compared with the 3′ end (+/−3 nt) (Additional file 1: Figure S8A and 8B). Of the 7183 unique reads, only 2210 reads (31.8%) have no variation on both the 5′ and 3′ ends (Additional file 1: Figure S8C). If we examine only one end, 5571 reads (63.64%) show 3′ end variations whereas only 1339 reads (18.64%) show 5′ end variations. The pattern of distributions shown in Additional file 1: Figure S8 is in accordance with miRNA’s biological role and biogenesis. It is believed the first 8 nucleotides are crucial for miRNA’s binding to its targeted mRNAs, and the variation at the 5′ end is therefore functionally more constrained. The larger variability at the 3′ end mainly results from imperfect Dicer editing, which either adds additional hairpin nucleotides or shortens the length of the miRNA, most commonly at the 3′ end. Argonaute crystallographic studies have indicated that the the 3′ ends extend from the PAZ domain and are therefore susceptible to exonucleolytic cleavage [42, 43], causing 3′ end shortening. Moreover, non-templated nucleotide addition to the 3′ end can occur on the mature miRNA [44]. Taken together, it is expected that the 3′ end of miRNA displays higher variation than the 5′ end.

### Rich and interactive visualization features in QuickMIRSeq

**Fig. 8:**
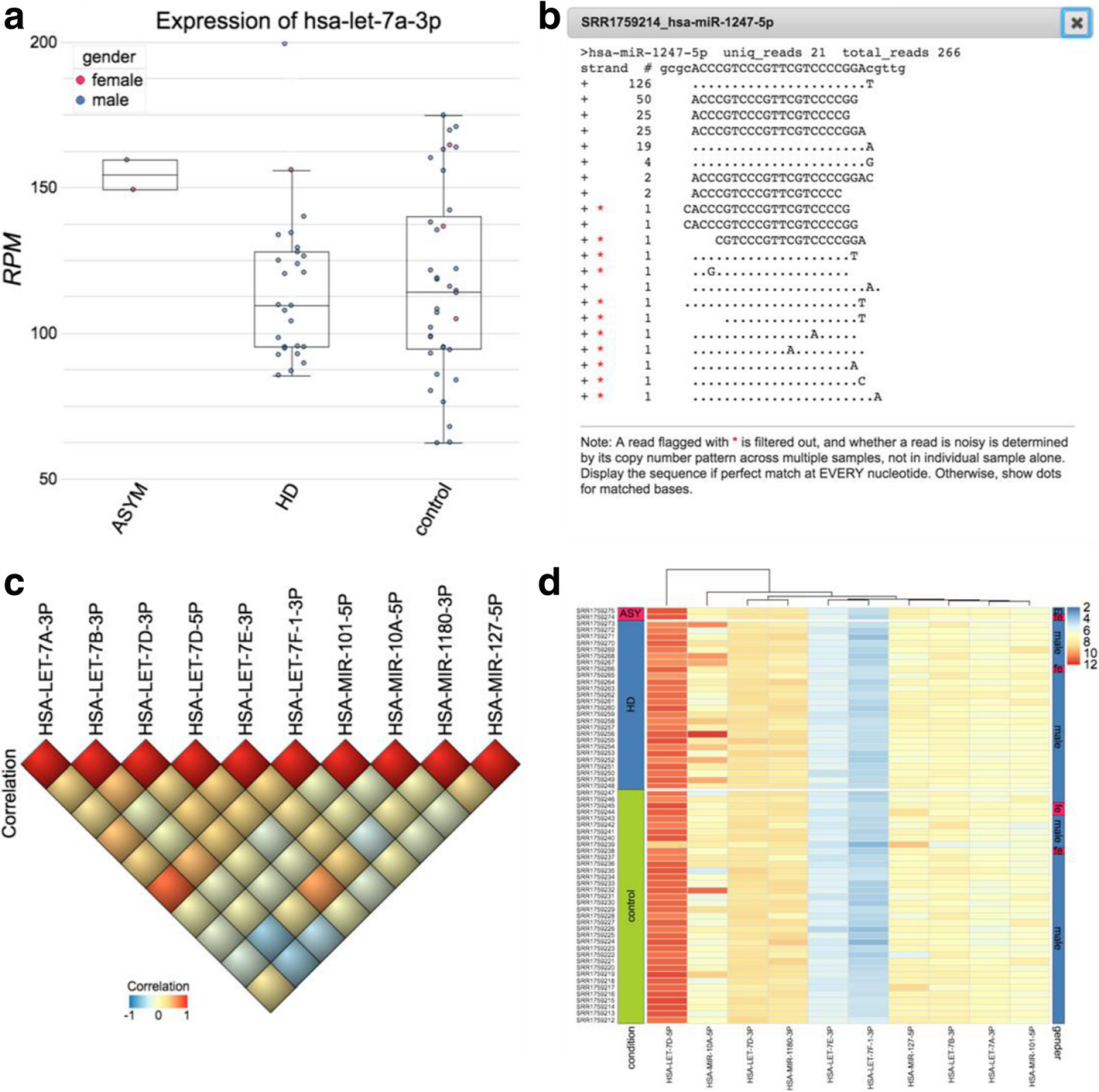
Rich interactive visualization features of QuickMIRSeq report. **a** Boxplot of expression values grouped by sample conditions. **b** Alignment details of reads mapped to has-miR-1247-5p. **c** Correlation plot of selected microRNA expressions. **d** Heatmap view of multiple microRNA expressions in log2 scale. Note all plots are generated interactively. The demo dataset is from GSE64977

The rich interactive features of QuickMIRSeq are partially illustrated in Fig. 8. As shown in Fig. 8a, miRNA expression profiles can be grouped and segregated in real time according to the sample annotations, such as time points, biological conditions and dosage arms. The look and feel of the plot is highly customizable including plot type, font size, color, and position of each box. The alignments of sequence reads with microRNA (Fig. 8b) show details of abundance of isomiRs, patterns of 5’ and 3’ offsets, and potential noisy reads. Correlation plot can be generated based on user selected microRNAs as shown in Fig. 8c to explore co-expression patterns. The heatmap shown in Fig. 8d is highly interactive. Data transformation and hierarchical and k-mean clustering can be performed through user menu. The QuickMIRSeq user guide (https://baohongz.github.io/guide/QuickMIRSeq.html) has detail instructions on how to explore the analysis results interactively.

### QuickMIRSeq versus miRge and other tools

The software miRge is one of the latest tools available for known miRNA quantification. It is considerably more computationally efficient than any prior software tools and scales well with large datasets [32]. The comparisons for samples SRR1759212, SRR1759213, SRR1759214, and SRR1759215 are shown in Additional file 1: Figure S9. The scatter plots indicate the quantification results are reasonably consistent for most miRNAs, but for some miRNAs, large differences were observed. The difference mainly results from the fact that miRge ignores strand information when analyzing miRNA datasets and that its execution workflow tends to exclude reads with mismatches from quantification, as discussed further in Additional file 1: Figure S9.

Many tools developed in early years are sort of superseded by recent ones. The new tools published in 2014 and 2015 included sRNAtoolbox [26], Oasis [29], iSRAP [30], CAP-miRSeq [31] and miRge [32]. The comparison of miRge with sRNAtoolbox was reported in the miRge paper. Oasis is a web based application and does not meet our needs. We installed iSRAP but failed to make it work. CAP-miRSeq was claimed to be a comprehensive analysis pipeline for microRNA sequencing data. In essence, CAP-miRSeq is a wrapper of miRDeep2 [23], and simplifies batch processing of multiple samples. However, in order to process multiple samples in parallel, CAP-miRSeq requires a Sun Grid Engine cluster. Unfortunately, Pfizer’s HPC cluster is running LSF. In contrast, QuickMIRSeq does not have special requirements on its execution environment, and can be run in a stand-alone Linux workstation or any cluster environment.

bcbio-nextgen also implements a configurable best-practices pipeline for small RNA-seq data analysis (https://bcbio-nextgen.readthedocs.io/en/latest/contents/pipelines.html#smallrna-seq), including quality controls, adapter trimming, miRNA/isomiR quantification, other small RNA detection, and prediction of new miRNAs. The quantification of known small RNAs is carried out by SeqBuster [45], a bioinformatic tool developed in 2010, while the quantification isomiRs is done by R script. In contrast, QuickMIRSeq quantifies both known miRNAs and isomiRs simultaneously. QuickMIRSeq is simple to use, and its implementation blends many useful features from other open source tools. More importantly, QuickMIRSeq makes all analysis results fully accessible via a web interface, and enables end users to visualize them interactively.

## Discussion

### QuickMIRSeq highlights

The analysis of miRNA-seq data presents unique challenges. The miRNA-seq dataset are intrinsically stranded, and QuickMIRSeq incorporates the strand information in the alignment step for more accurate quantification (Fig. 5). Compared with mRNA-seq, miRNA sequences are typically only 19–23 bp in length, and are more likely to be mapped to random sequences throughout the genome. To remedy this situation, QuickMIRSeq introduces joint mapping of multiple samples not only to reduce computational time (Fig. 6a), but also to filter out potentially false positives (i.e., noisy reads) based upon their expression patterns across samples, thereby improving the reliability of the detected miRNAs (Fig. 6b). Additionally, QuickMIRSeq introduces the step of remapping reads with mismatches to a reference genome to further reduce the number of false hits (Fig. 6c). The combined effect of our approaches maximizes the capture of true miRNAs and minimizes false assignments. Besides, QuickMIRSeq quantifies the expression levels for both miRNAs and isomiRs simultaneously.

QuickMIRSeq automatically generates a rich set of QC metrics and publication-ready plots and a variety of summary tables (Figs. 4 and 8). The summary plots on adapter trimming as well as the read length distribution offer concise information on the quality of the raw dataset. After alignment, various plots, including the number of detected miRNAs, the distribution of annotated sequences and the read redundancy in each annotated category (Fig. 7) can be used to quickly uncover potential sequencing issues in some samples or in the entire dataset, such as primer dimerization or sample preparation failures. The rich visualization features implemented in QuickMIRSeq allow end users to interactively explore the results of miRNA-seq data analyses, and to gain more insights into miRNA-seq datasets without setting up database and/or server.

QuickMIRSeq is very easy to use. For practical miRNA-seq data analysis, a user just needs to prepare *run.config*, a plain text configuration file that stores project, species, and software-specific parameters, such as the location of database and sequencing files. This run configuration file also improves the reproducibility of miRNA-seq data analyses. For the convenience of QuickMIRSeq users, a configuration file template has been provided in the QuickMIRSeq package for easy customization. Furthermore, end users have full control of the execution workflow in Step #2 by enabling or disabling some optional computational analysis steps. Step #3 does not require any parameters when running the *QuickMIRSeq-report.pl* script under the results folder.

### Limitations of the QuickMIRSeq pipeline

QuickMIRSeq is designed for accurate quantification of known miRNAs and isomiRs. The current version of QuickMIRSeq cannot be used to discover novel miRNAs. The counts table generated from QuickMIRSeq provides a starting point for functional analysis and biological interpretation. Downstream analyses are usually driven by biological questions and the experimental design, and thus vary from project to project. Currently, QuickMIRSeq cannot be used to perform differential expression analysis of miRNAs [46, 47] or carry out gene set overrepresentation analysis [48]. We attempted to automate differential analysis but realized it is extremely hard to make this step user friendly and universally applicable to any experimental design. Some tools like iSRAP [30] and CAP-miRSeq [31] offer such a functionality, but support only the comparison between two conditions such as “Treatment versus Control”. A practical microRNA-seq study is quite often much more complex. For instance, for biomarker discovery in clinical studies, it’s common to collect specimens at different time points, from different population (race, gender or age group), from various disease stages or treatment arms (healthy control, disease subgroups, and different dosage group), and even from different sources (whole blood, PBMC, urine or tissues). As a result, the statistical model and covariates for differential analysis can be very complicated. The “Treatment versus Control” comparison is too simple to be practically used in most miRNA-seq data analysis.

Another limitation is sample size. QuickMIRSeq collapses sequences into unique sequences, first within and across samples, and then annotates them. All unique reads and their quantification are held in memory; therefore, the number of samples that can be run together is not unlimited. The read counts table is kept in memory, and its size and growth is roughly proportional to the number of unique reads. If the miRNA-seq process is clean and the majority of reads are miRNAs, the table should not grow significantly as each sample is added. According to our internal test runs, QuickMIRSeq should have no problem in batching 200 samples on a Linux workstation with 128 GB memory. Until now, the majority of miRNA-seq datasets deposited into GEO have sample sizes much smaller than 100. In case the samples from a large-scale miRNA-seq study cannot be processed in one batch by QuickMIRSeq, the large dataset can be divided into multiple chunks for parallel processing.

## Conclusion

We developed QuickMIRSeq, an integrated pipeline for quick and accurate quantification of known miRNAs and isomiRs by jointly processing multiple samples. Its implementation takes advantage of the unique nature of miRNAs, and is computationally efficient. A variety of strategies have been introduced to maximize the capture of true miRNAs, to minimize false positives, and to improve the reliability of miRNA detection and quantification. The user-friendly interactive application makes data exploration and sharing more efficient.

## Additional files

Additional file 1: Table S1. Human mature miRNAs in miRBase Release 21 with identical sequences. **Table S2**. Human hairpins in miRBase Release 21 with identical sequences. **Table S3**. Pairs of miRNAs that are reverse complementary to each other in human miRBase Release 21. **Table S4**. Top 10 miRNAs with large differences in miRNA quantification between stranded and non-stranded mapping modes. **Table S5**. Distribution of 5′ and 3′ end offsets of unique miRNA reads in GSE64977. **Figure S1**. Top panel: All of the miRNAs in the alignment have the same mature sequence (highlighted in gray), but originate from different genes as evidenced by the differences in the pre-miRNA sequences. Bottom panel: miRNA genes found in a cluster on human chromosome 19. **Figure S2**. Protocol of isomiR quantification. **Figure S3**. Scatter plots of miRNA quantification results by miRge for samples SRR1759212 SRR1759213, SRR1759214 and SRR1759215. The same dataset were analyzed with and without incorporation of the strand information, respectively. **Figure S4**. Breakdown of mapped miRNA reads into perfect and mismatch categories. **Figure S5**. Comprehensive annotation of miRNA-seq reads. The summary plot provides an overview of the distribution of annotated reads in all five annotated RNA categories for each sample. **Figure S6**. Summary report for adapter trimming. **Figure S7**. Read length distributions for samples SRR1759212, SRR1759213, SRR1759214, and SRR1759215 in the GSE64977 miRNA-seq dataset. **Figure S8**. Variations at 5′ and 3′ ends of miRNA reads. **Figure S9**. The comparison of QuickMIRSeq with miRge. (PDF 1088 kb)

## Abbreviations

isomiRs: MiRNA isoforms
miRISC: MiRNA-induced silencing complex
miRNA: MicroRNA
NGS: Next generation sequencing
Nts: Nucleotides
QC: Quality control
