# Supplementary material for: QuickMIRSeq: a pipeline for quick and accurate quantification of both known miRNAs and isomiRs by jointly processing multiple samples from microRNA sequencing
Source: BMC Bioinformatics. 2017 Mar 20;18:180. doi: 10.1186/s12859-017-1601-4 (PMC5359966; doi:10.1186/s12859-017-1601-4)
Supplement: Additional file 1: Table S1. — Human mature miRNAs in miRBase Release 21 with identical sequences. Table S2. Human hairpins in miRBase Release 21 with identical sequences. Table S3. Pairs of miRNAs that are reverse complementary to each other in human miRBase Release 21. Table S4. Top 10 miRNAs with large differences in miRNA quantification between stranded and non-stranded mapping modes. Table S5. Distribution of 5′ and 3′ end offsets of unique miRNA reads in GSE64977. Figure S1. Top panel: All of the miRNAs in the alignment have the same mature sequence (highlighted in gray), but originate from different genes as evidenced by the differences in the pre-miRNA sequences. Bottom panel: miRNA genes found in a cluster on human chromosome 19. Figure S2. Protocol of isomiR quantification. Figure S3. Scatter plots of miRNA quantification results by miRge for samples SRR1759212 SRR1759213, SRR1759214 and SRR1759215. The same dataset were analyzed with and without incorporation of the strand information, respectively. Figure S4. Breakdown of mapped miRNA reads into perfect and mismatch categories. Figure S5. Comprehensive annotation of miRNA-seq reads. The summary plot provides an overview of the distribution of annotated reads in all five annotated RNA categories for each sample. Figure S6. Summary report for adapter trimming. Figure S7. Read length distributions for samples SRR1759212, SRR1759213, SRR1759214, and SRR1759215 in the GSE64977 miRNA-seq dataset. Figure S8. Variations at 5′ and 3′ ends of miRNA reads. Figure S9. The comparison of QuickMIRSeq with miRge. (PDF 1088 kb) [file 12859_2017_1601_MOESM1_ESM.pdf]

Title: QuickMIRSeq lifts joint quantification of known miRNAs and isomiRs from multiple samples to the next level of automation and interactive visualization

Table S1. Human miRNAs in miRBase Release 21 with identical sequences

1.	hsa-miR-365b-3p	hsa-miR-365a-3p				
2.	hsa-miR-548z	hsa-miR-548h-3p				
3.	hsa-miR-3689c	hsa-miR-3689b-3p				
4.	hsa-miR-518a-5p	hsa-miR-527				
5.	hsa-miR-570-5p	hsa-miR-548ai				
6.	hsa-miR-523-5p	hsa-miR-519b-5p	hsa-miR-522-5p	hsa-miR-519c-5p	hsa-miR-518e-5p	hsa-miR-519a-5p
7.	hsa-miR-3689b-5p	hsa-miR-3689e	hsa-miR-3689a-5p			
8.	hsa-miR-548ad-5p	hsa-miR-548ae-5p				
9.	hsa-miR-199b-3p	hsa-miR-199a-3p				
10.	hsa-miR-518d-5p	hsa-miR-526a	hsa-miR-520c-5p			
11.	hsa-miR-548t-3p	hsa-miR-548aa				
12.	hsa-miR-548am-5p	hsa-miR-548c-5p	hsa-miR-548o-5p			
13.	hsa-miR-517b-3p	hsa-miR-517a-3p				
14.	hsa-miR-548aj-5p	hsa-miR-548x-5p	hsa-miR-548g-5p			
15.	hsa-miR-516b-3p	hsa-miR-516a-3p				

Table S2. Human hairpins in miRBase Release 21 with identical sequences

1.	hsa-mir-941-3	hsa-mir-941-5	hsa-mir-941-2	hsa-mir-941-4
2.	hsa-mir-6511a-2	hsa-mir-6511a-3	hsa-mir-6511a-1	hsa-mir-6511a-4
3.	hsa-mir-4650-2	hsa-mir-4650-1		
4.	hsa-mir-3690-2	hsa-mir-3690-1		
5.	hsa-mir-515-2	hsa-mir-515-1		
6.	hsa-mir-1302-11	hsa-mir-1302-10	hsa-mir-1302-9	hsa-mir-1302-2
7.	hsa-mir-4771-2	hsa-mir-4771-1		
8.	hsa-mir-4283-2	hsa-mir-4283-1		
9.	hsa-mir-3118-4	hsa-mir-3118-3	hsa-mir-3118-2	
10.	hsa-mir-3648-2	hsa-mir-3648-1		
11.	hsa-mir-1972-2	hsa-mir-1972-1		
12.	hsa-mir-3180-3	hsa-mir-3180-1		
13.	hsa-mir-514a-2	hsa-mir-514a-3		
14.	hsa-mir-6770-2	hsa-mir-6770-3	hsa-mir-6770-1	
15.	hsa-mir-4315-1	hsa-mir-4315-2		
16.	hsa-mir-6089-1	hsa-mir-6089-2		
17.	hsa-mir-8071-1	hsa-mir-8071-2		
18.	hsa-mir-3670-3	hsa-mir-3670-4	hsa-mir-3670-1	hsa-mir-3670-2
19.	hsa-mir-4509-3	hsa-mir-4509-1	hsa-mir-4509-2	
20.	hsa-mir-5701-1	hsa-mir-5701-2	hsa-mir-5701-3	
21.	hsa-mir-1244-2	hsa-mir-1244-3	hsa-mir-1244-1	hsa-mir-1244-4
22.	hsa-mir-1233-2	hsa-mir-1233-1		
23.	hsa-mir-6724-3	hsa-mir-6724-4	hsa-mir-6724-2	hsa-mir-6724-1
24.	hsa-mir-6862-1	hsa-mir-6862-2		
25.	hsa-mir-8069-1	hsa-mir-8069-2		
26.	hsa-mir-3687-1	hsa-mir-3687-2		
27.	hsa-mir-3680-2	hsa-mir-3680-1		
28.	hsa-mir-3179-2	hsa-mir-3179-1	hsa-mir-3179-3	hsa-mir-3179-4
29.	hsa-mir-4436b-1	hsa-mir-4436b-2		
30.	hsa-mir-4444-2	hsa-mir-4444-1		
31.	hsa-mir-6859-3	hsa-mir-6859-4	hsa-mir-6859-2	hsa-mir-6859-1
32.	hsa-mir-1184-1	hsa-mir-1184-3	hsa-mir-1184-2	

Table S3. miRNA pairs that are reverse complementary to each other

1.	hsa-miR-550a-3p	hsa-miR-550b-2-5p
2.	hsa-miR-103b	hsa-miR-103a-3p
3.	hsa-miR-423-5p	hsa-miR-3184-3p
4.	hsa-miR-3913-3p	hsa-miR-3913-5p
5.	hsa-miR-4670-5p	hsa-miR-4670-3p
6.	hsa-miR-374b-5p	hsa-miR-374c-3p
7.	hsa-miR-642b-5p	hsa-miR-642a-3p
8.	hsa-miR-5591-5p	hsa-miR-5591-3p
9.	hsa-miR-4433b-5p	hsa-miR-4433a-3p

Table S4. Top 10 miRNAs with differences in miRNA quantification between stranded and non-stranded mapping modes (Dataset: GSE64977. The value in each cell represents the number of reads mapped to the corresponding miRNA.)

miRNA	Stranded mapping				Nonstranded mapping			
	SRR1759212	SRR1759213	SRR1759214	SRR1759215	SRR1759212	SRR1759213	SRR1759214	SRR1759215
hsa-miR-103b	0	0	0	0	7523	9956	7456	7516
hsa-miR-486-3p	5	19	15	12	1562	4388	5124	2660
hsa-miR-3065-5p	59	66	58	19	1650	4082	3129	2861
hsa-miR-3074-5p	1	3	5	1	1539	2591	1584	1655
hsa-miR-3184-5p	0	0	0	0	1239	2337	1947	1159
hsa-miR-219b-5p	2	4	3	4	661	1631	1171	1149
hsa-miR-219b-3p	2	4	4	2	436	1208	981	1066
hsa-miR-3065-3p	83	96	102	52	160	749	234	526
hsa-miR-3184-3p	0	0	0	0	59	239	169	197
hsa-miR-203b-5p	0	0	0	2	79	114	82	70

Table S5. Distribution of 5´ and 3´ end offsets of unique miRNA reads in GSE64977

n/a	3' End										Sum	
5' End	**Offset**	**-4**	**-3**	**-2**	**-1**	**0**	**1**	**2**	**3**	**4**	**5**	**Count**	**%**
	**-3**	0	0	5	8	17	13	12	10	6	0	71	0.99
	**-2**	1	1	9	16	35	41	51	19	4	0	177	2.46
	**-1**	5	12	35	63	161	139	94	26	5	1	541	7.53
	**0**	126	260	562	1066	2210	936	541	108	29	6	5844	81.36
	**1**	12	25	70	103	162	49	10	0	1	0	432	6.01
	**2**	6	14	19	25	23	7	2	0	0	0	96	1.34
	**3**	4	6	3	4	3	0	0	0	0	0	20	0.28
	**4**	1	0	0	0	1	0	0	0	0	0	2	0.03
**Sum**	**Count**	155	318	703	1285	2612	1185	710	163	45	7	**7183**	100
	**%**	2.16	4.43	9.79	17.89	36.36	16.50	9.88	2.27	0.63	0.10	100	n/a

```
hsa-miR-518e-5p                    CUCUAGAGGGAAGCGCUUUCUG
hsa-mir-518e     UCUCAGGCUGUGACCCUCUAGAGGGAAGCGCUUUCUGUUGGCUAAAAGAAAAGAAAGCGCUUCCCUUCAGAGUGUUAACGCUUUGAGA
hsa-mir-523      UCUCAUGCUGUGACCCUCUAGAGGGAAGCGCUUUCUGUUGUCUGAAAGAAAAGAACGCGCUUCCCUAUAGAGGGUUACCCUUUGAGA
hsa-mir-519a      CUCAGGCUGUGACACUCUAGAGGGAAGCGCUUUCUGUUGUCUGAAAGAAAGGAAAGUGCAUCCUUUUAGAGUGUUACUGUUUGAG
hsa-mir-519b        CAUGCUGUGACCCUCUAGAGGGAAGCGCUUUCUGUUGUCUGAAAGAAAAGAAAGUGCAUCCUUUUAGAGGUUUACUGUUUG
hsa-mir-519c     UCUCAGCCUGUGACCCUCUAGAGGGAAGCGCUUUCUGUUGUCUGAAAGAAAAGAAAGUGCAUCUUUUUAGAGGAUUACAGUUUGAGA
hsa-mir-522      UCUCAGGCUGUGUCCCUCUAGAGGGAAGCGCUUUCUGUUGUCUGAAAGAAAAGAAAAUGGUUCCCUUUAGAGUGUUACGCUUUGAGA
```

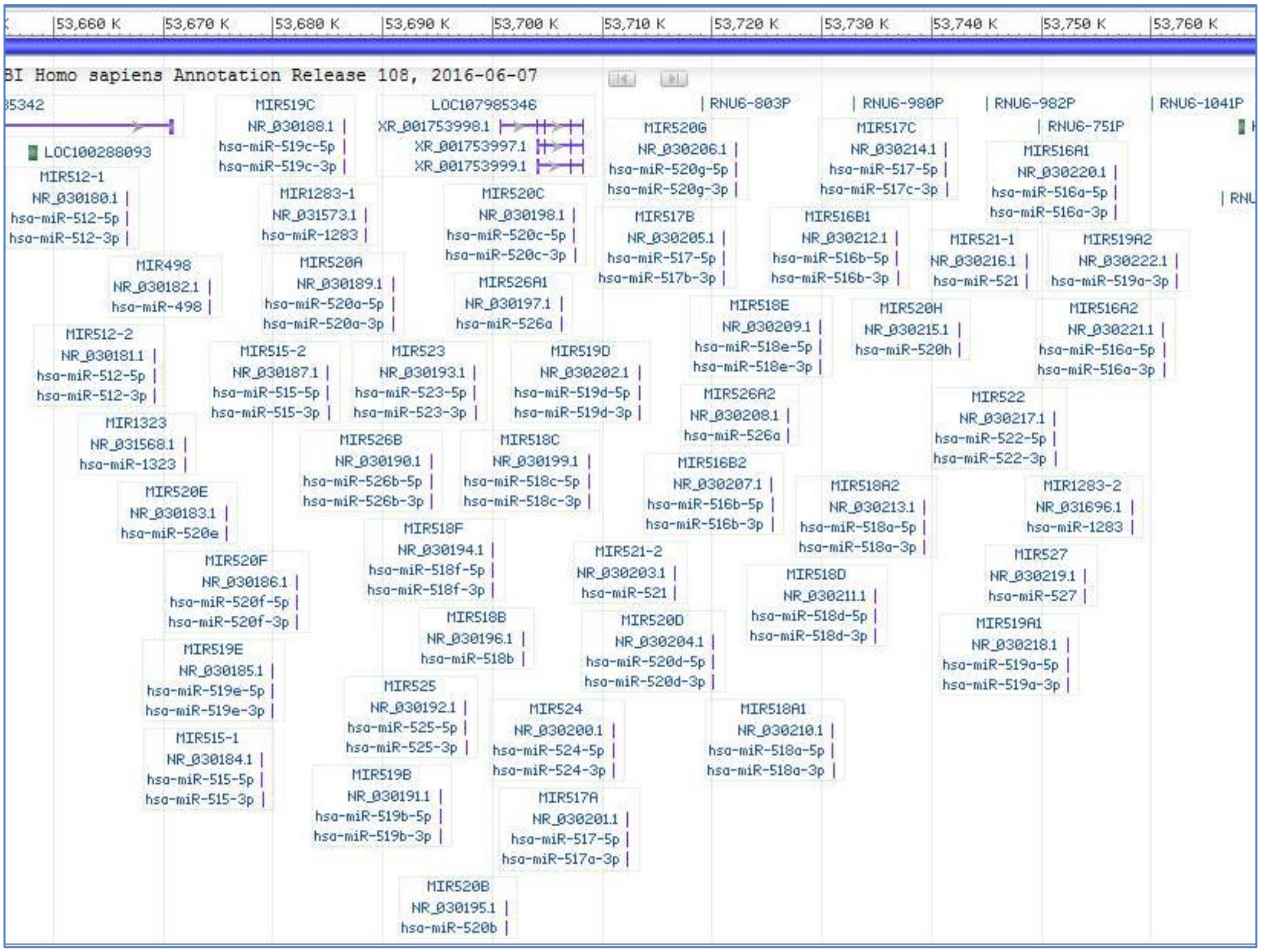

Figure S1. Top panel: All of the miRNAs in the alignment have the same mature sequence (highlighted in gray). However, they originate from different genes as evidenced by the differences in the pre-miRNA sequences. **Bottom panel:** Genes found in a cluster on human chromosome 19. A single gene may have expanded into all the other paralogs, while maintaining the same mature miRNA sequence but undergoing divergence in the genomic sequence, albeit slowly.

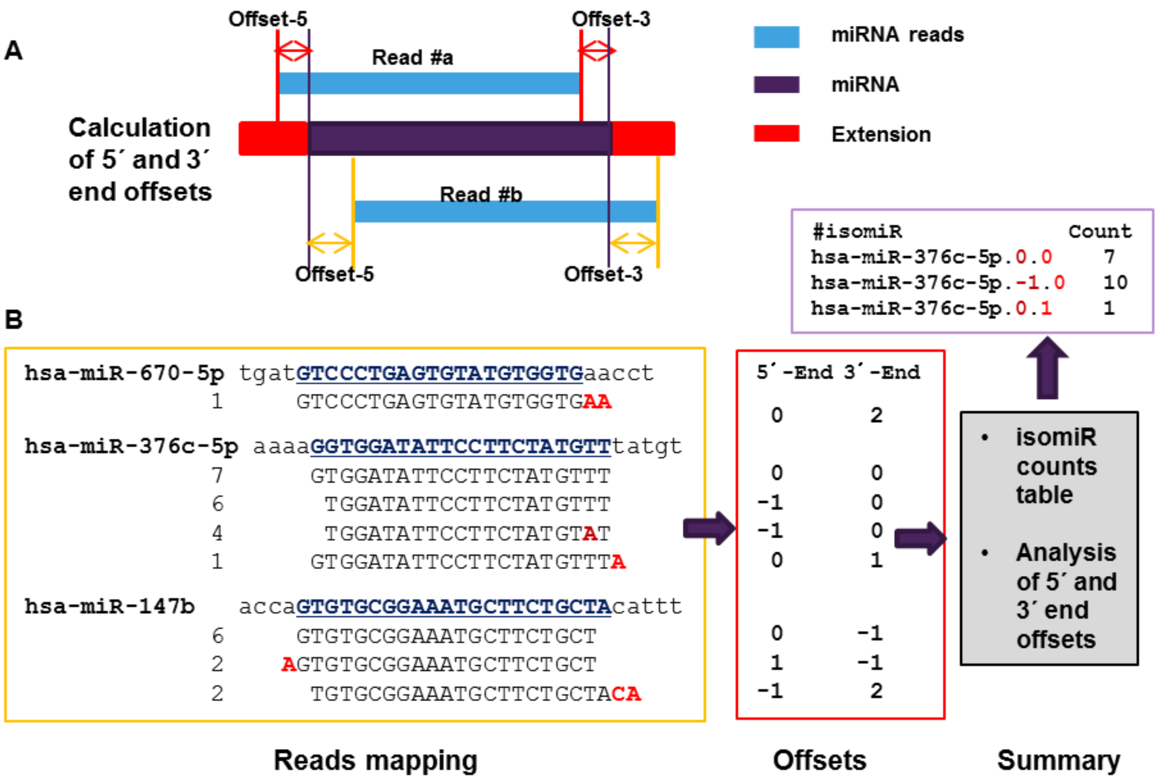

Figure S2. Protocol of isomiR quantification. A) Definition of the 5´ and 3´ end offsets. B) Isoform quantification. For all unique reads, the 5´ and 3´ end offsets are calculated first. Next, reads that have identical 5´ and 3´ end offsets are added up to generate an isomiR counts table. Each isomiR in the counts table is denoted as *miRNA.5´end-offset.3´end-offset*, for instance, *hsa-miR-376c-5p.−1.0*.

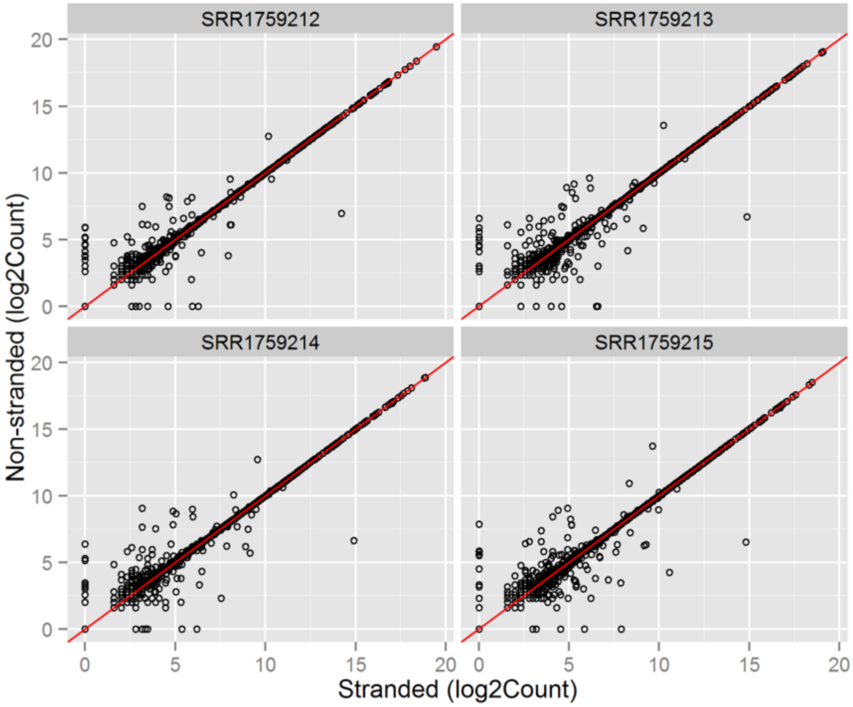

Figure S3. Scatter plots of miRNA quantification results by miRge for samples SRR1759212 SRR1759213, SRR1759214 and SRR1759215. The miRNA-seq dataset for all 4 samples were stranded, and analyzed using miRge with and without incorporation of the strand information, respectively. It is noted that the miRge does not take the strand information into account in the alignment step. The original source code of miRge was modified to incorporate the strand information into miRNA-seq data analysis.

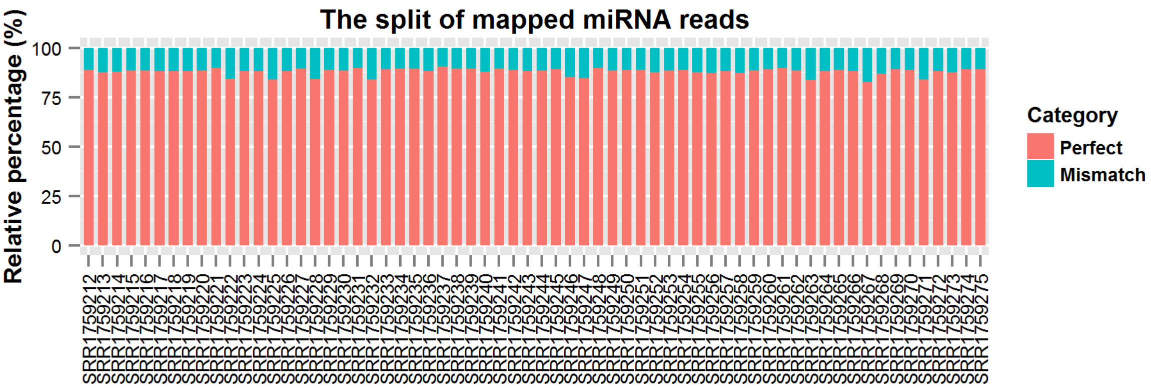

Figure S4. Breakdown of mapped miRNA reads. On average, about 12% (ranging from 10 to 17%) mapped reads have mismatches in a sample. The sample names from the GSE64977 miRNA-seq dataset used in this study are shown along the x-axis.

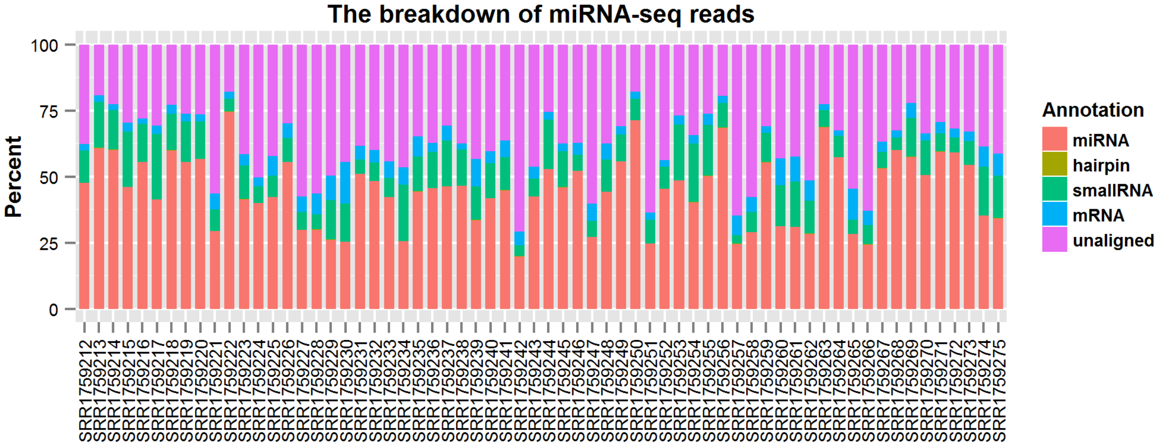

Figure S5. Comprehensive annotation of miRNA-seq reads. The summary plot provides an overview of the distribution of annotated reads in all five annotated RNA categories for each sample. The sample names from the GSE64977 miRNA-seq dataset used in this study are shown along the x-axis.

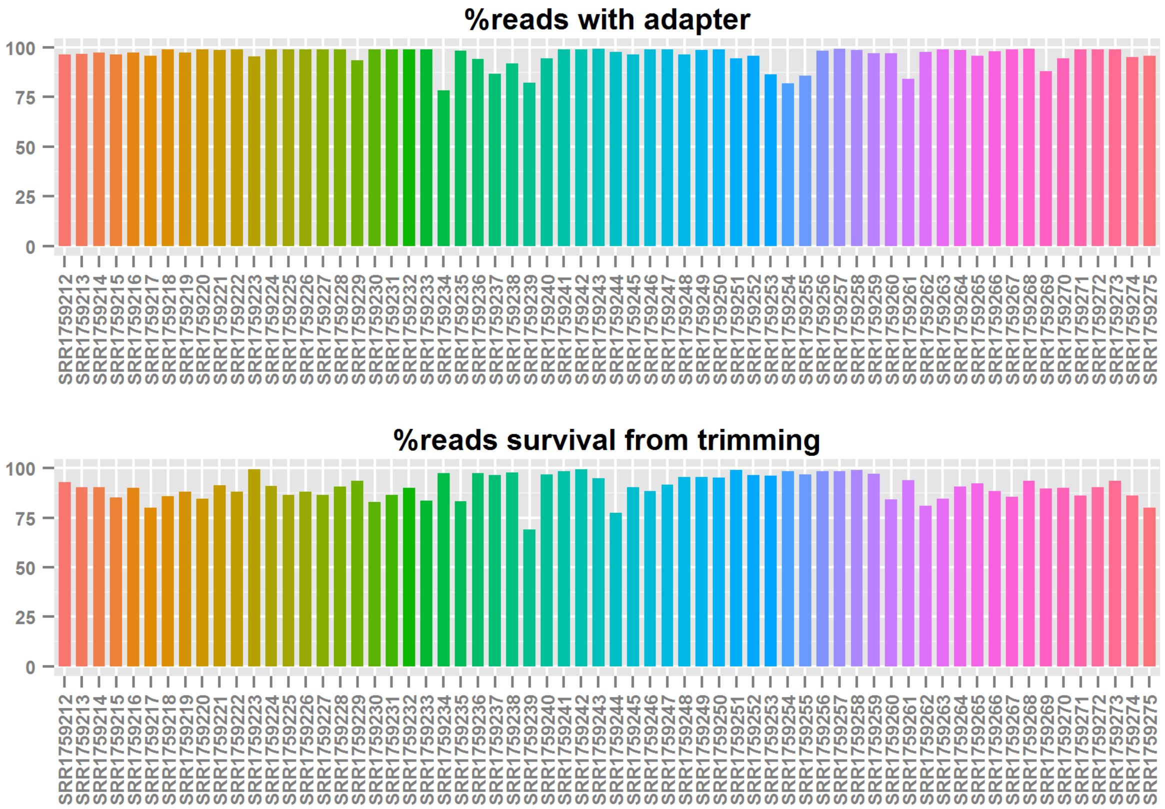

Figure S6. Summary report for adapter trimming. Ideally, the percentage of reads with adapter sequences should be close to 100% in a high-quality miRNA-seq dataset (**Top panel**). The trimmed reads should still be long enough to be kept for alignment, and thus, the percentage of reads surviving adapter trimming should also be close to 100% (**Bottom panel**). (The samples are from GSE64977, a stranded miRNA-seq dataset.)

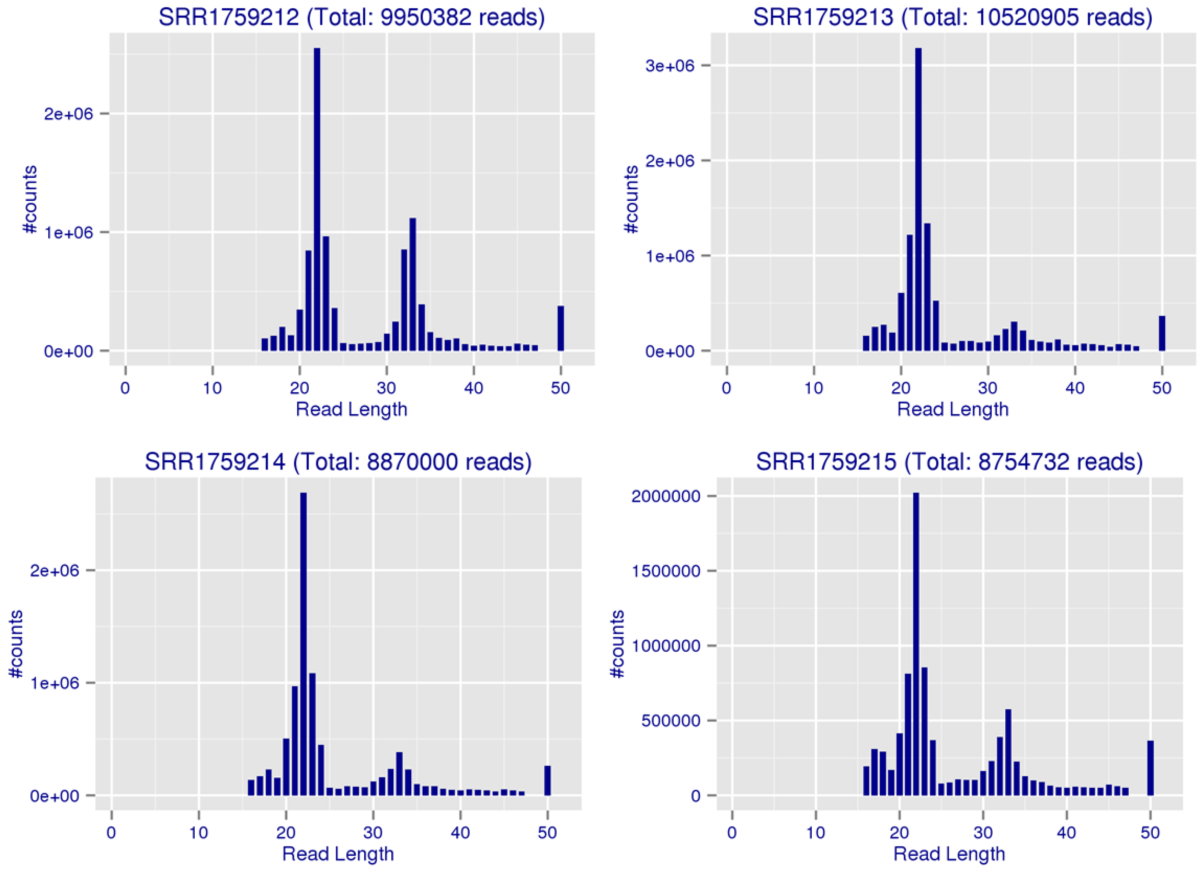

Figure S7. Read length distributions for samples SRR1759212, SRR1759213, SRR1759214, and SRR1759215 in the GSE64977 miRNA-seq dataset. The length distribution of the adaptor-trimmed reads is, as expected, centered on 22 bp. The overall pattern of distribution indicates miRNA sequencing is reliable and the majority of reads are derived from miRNAs.

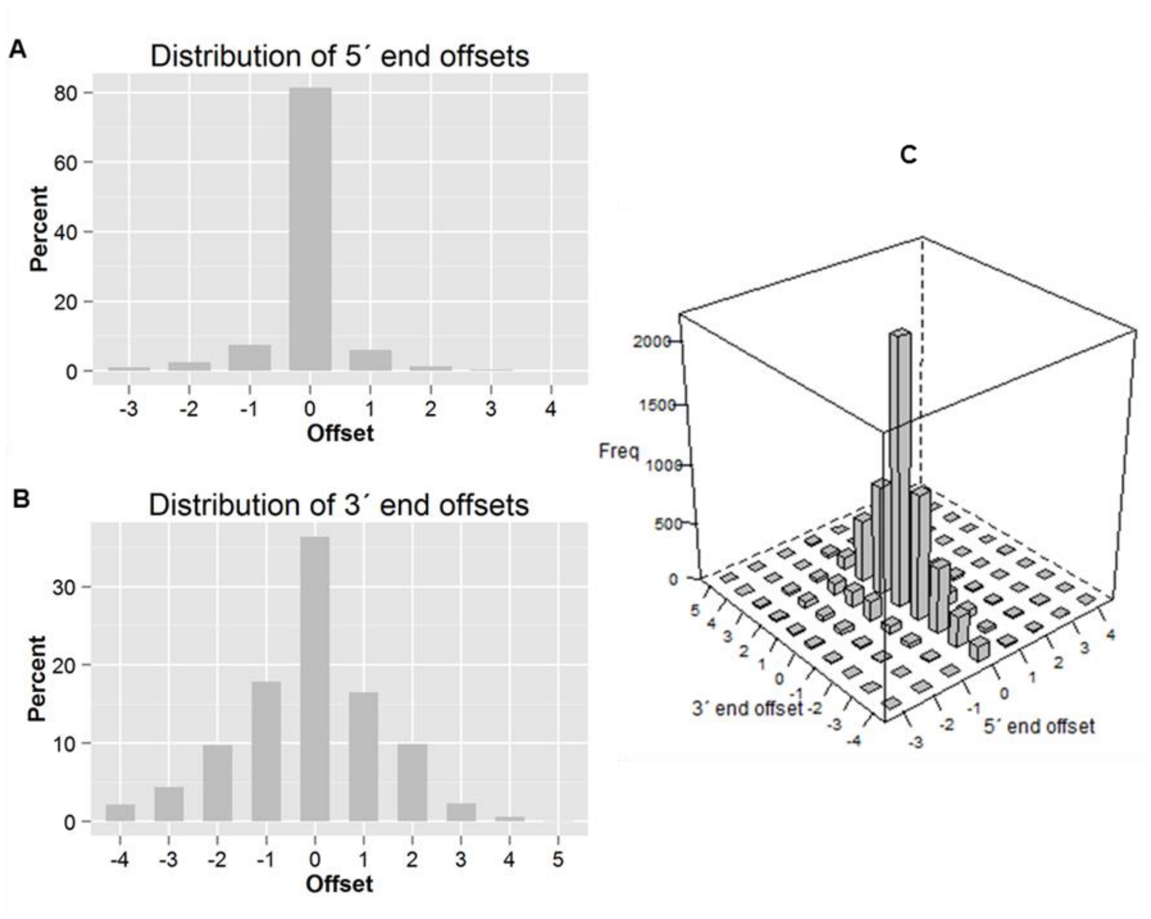

Figure S8. Variations at 5´ and 3´ ends of miRNA reads. A, B) Distribution of offsets for the 5´ and 3´ ends. C) Joint distribution of variations at the 5´ and 3´ ends. Of the 7183 unique reads, only 2210 reads (31.8%) have no variation at all on both the 5´ and 3´ ends; 5571 reads (63.64%) show 3´-end variations whereas only 1339 reads (18.64%) show 5´-end variations. Generally speaking, the 5´ end is more conserved and shows a much narrower range (+/− 1 nt) of variations compared with the 3´ end (+/− 3 nts). (The results were from GSE64977 miRNA-seq dataset.)

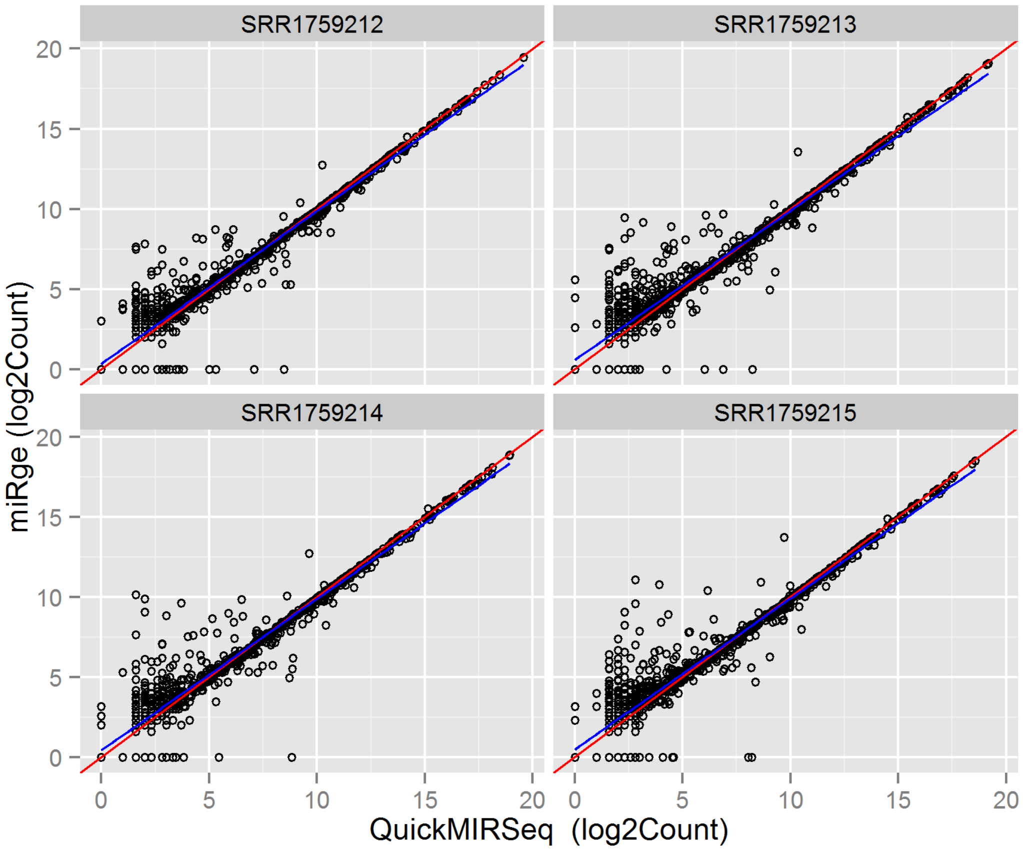

Figure S9. QuickMIRSeq versus miRge. The scatter plots indicate the quantification results are, in general, reasonably consistent for most miRNAs. However, large differences are observed for some miRNAs. The dots above the diagonal line at the bottom left corner tend to indicate large differences. The alignments indicate many reads in miRge are mistakenly mapped to antisense strands of miRNAs. The red line is the diagonal line and the blue line indicates a smooth linear regression.

Description of the miRge annotation process:

In miRge, there are two rounds of mapping to known miRNAs separated by three sequential mapping steps to hairpins, small RNAs, and mRNAs. In the first round mapping, no mismatch is allowed, while in the second round, up to three mismatches are allowed. Obviously reads with one mismatch will not be mapped in the first round. In miRge, such reads will not have a second chance to be mapped to miRNAs because they will be mapped to hairpins (miRNA precursors) in the sequential step that follows the first round mapping. As shown in Figure S5, an average 12% of sequences that should have been mapped to miRNAs are excluded from quantification in miRge. Moreover, the second round mapping in miRge can give rise to many potential false positives because up to three mismatches are allowed. This criterion is too loose considering miRNA sequences are very short.

Further, miRge allows reads to map to any strand of miRNA, regardless of the sequencing protocol. Consequently, high false positive rates are expected. The combined net effect is that miRge will capture fewer reads but report more false positives than QuickMIRSeq. The total mapped miRNA reads in the

miRge run are 4351703 (SRR1759212), 5816847 (SRR1759213), 4864378 (SRR1759214), and 3707141 (SRR1759215). The corresponding numbers in the QuickMIRSeq run are 4744589, 6399322, 5336200, and 4031699. Accordingly, the ratios of mapped miRNA reads are 0.917, 0.909, 0.912, and 0.919, which is consistent among samples. On average, QuickMIRSeq captures 8–9% more reads than miRge, but fewer false positives. The miRNAs at the bottom left corner but above the diagonal line in Figure S10 tend to have many reads mapped to their antisense strands by miRge because miRge ignores strand information and consequently reports more false positives than QuickMIRSeq.
